# Targeting Both Monomer and Oligomer with Fibrinogen Efficiently Suppresses Amyloid Fibril Formation and Cell Toxicity of Amyloid β 1‑42

**DOI:** 10.1021/acschemneuro.5c00562

**Published:** 2025-09-05

**Authors:** Naoki Yamamoto, Keisuke Yuzu, Ken Morishima, Rintaro Inoue, Masaaki Sugiyama, Daisuke Koyama, Eri Chatani

**Affiliations:** † Division of Biophysics, Physiology, School of Medicine, 12838 Jichi Medical University, 3311-1 Yakushiji, Shimotsuke, Tochigi 329-0498, Japan; ‡ Graduate School of Science, 163670 Kobe University, 1-1 Rokkodai-cho, Nada-ku, Kobe 657-8501, Japan; § Institute for Integrated Radiation and Nuclear Science, 12918 Kyoto University, 2 Asashiro-Nishi, Kumatori, Sennan-gun, Osaka 590-0494, Japan; ∥ Department of Hematology, 12775 Fukushima Medical University, Fukushima 960-1295, Japan

**Keywords:** amyloid β 1-42, Alzheimer’s disease, oligomer, fibrinogen, amyloid fibril, cell toxicity

## Abstract

The development of drugs for Alzheimer’s disease, which accounts for over half of all dementia cases, remains challenging. Amyloid β 1-42 (Aβ42) is widely recognized for its deposition in the brains of patients with Alzheimer’s disease. Furthermore, Aβ42-induced cell toxicity likely plays a role in disease onset. Molecular species present in the early stages, such as monomers and oligomers, are appropriate therapeutic targets for suppressing amyloid fibril formation and cell toxicity. In this study, we investigated the effects of bovine fibrinogen (bFg) and human fibrinogen (hFg) since these molecules have been known to exhibit chaperone-like activities. Our findings indicate that bFg exerts a strong inhibitory effect on amyloid fibril formation. Dot blot assays, analytical ultracentrifugation (AUC), and atomic force microscopy (AFM) suggest that bFg interacts with both Aβ42 monomers and oligomers. In contrast, human fibrinogen (hFg), which interacts only with oligomers, exhibits a weaker inhibitory effect on amyloid fibril formation. Moreover, bFg significantly rescued cells from Aβ42-induced toxicity, whereas hFg provided only partial protection. These findings underscore the potential of molecules targeting early stage Aβ42 species as promising candidates for Alzheimer’s disease treatment.

## Graphical abstract

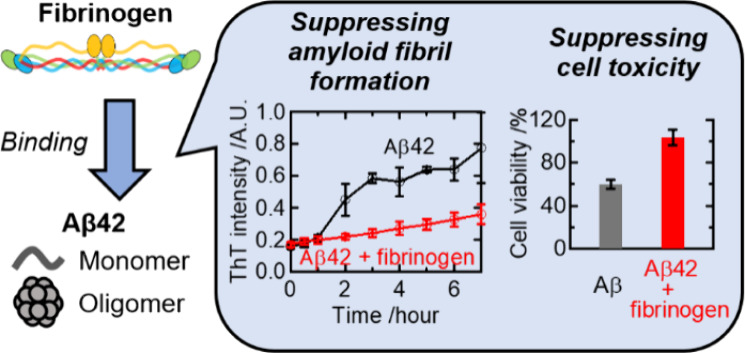

## Introduction

Alzheimer’s disease is the leading cause of dementia, affecting millions of people worldwide. Developing therapeutic strategies for this disease is an urgent global priority, particularly given the aging population. Recently, the first therapeutic drug, Lecanemab, was launched and has been shown to prevent cognitive decline.^1,2^ However, its use is limited to the early stages of dementia or mild cognitive impairment (MCI), highlighting the need for additional therapeutic options.

Amyloid β 1-42 (Aβ42) forms amyloid fibrils that accumulate in the brain, leading to the formation of senile plaques. The presence of these plaques is thought to contribute to the development of neurofibrillary tangles, in which amyloid fibrils composed of phosphorylated tau protein accumulate in the nervous system, ultimately resulting in neuronal dysfunction.^3^ Therefore, preventing Aβ42 amyloid fibril formation is one strategy to mitigate the risk of Alzheimer’s disease. Since amyloid fibrils form through the polymerization of molecular species present in the early stages, such as monomers and oligomers,^4^ inhibitors that block this transition could serve as a viable therapeutic approach. For example, rosmarinic acid has been shown to suppress Aβ42 amyloid fibril formation.^5^

In addition to fibril formation, Aβ42 monomers and oligomers may also contribute to Alzheimer’s disease by exerting cytotoxic effects. For example, monomeric Aβ42 is known to form channels in the lipid bilayer.^6^ Furthermore, Aβ42 oligomers induce synaptic dysfunction by damaging cell membranes.^7^ Molecules that interact with Aβ42 monomers or oligomers may mitigate these effects. For instance, rosmarinic acid reduces cytotoxicity by binding to Aβ42 monomers.^8^ These findings suggest that molecules capable of interacting with Aβ42 monomers or oligomers are promising candidates for preventing Alzheimer’s disease by inhibiting both amyloid fibril formation and Aβ42-induced cytotoxicity.

In the proteostasis system, molecular chaperones in the cytosol or extracellular regions inherently prevent amyloid fibril formation and mitigate cell toxicity.^9^ For example, heat shock proteins (HSPs) 70 and 90, which function in the cytosol, suppress Aβ42 amyloid fibril formation.^10,11^ Clusterin, present in extracellular fluids, inhibits Aβ42 amyloid fibril formation by interacting with oligomers and also reduces cell toxicity.^12^ In addition to these molecular chaperones, some proteins exhibit chaperone activity despite having primary functions unrelated to chaperoning. These proteins are referred to as chaperone-like proteins. Fibrinogen, a blood-clotting protein universally present in plasma and conserved across vertebrates, is a well-known chaperone-like protein.^13–15^ It has a molecular weight of approximately 340 kDa and a rod-like structure composed of six polypeptide chains (two Aα, two Bβ, and two γ chains).^16^ Human fibrinogen (hFg) has been shown to suppress amyloid fibril formation of several amyloid-prone proteins.^17,18^ Similarly, bovine fibrinogen (bFg) interacts with molecular species that arise before amyloid fibril formation, preventing the structural transitions necessary for fibrillization.^19,20^ Based on the facts, we speculated that these fibrinogen molecules are effective on suppressing amyloid fibril formation, which remains unclear.

In this study, we investigated whether fibrinogen molecules also suppress Aβ42 amyloid fibril formation. We demonstrated that bFg inhibits Aβ42 amyloid fibril formation. Using dot blot assays, analytical ultracentrifugation (AUC), and atomic force microscopy (AFM), we showed that bFg interacts with Aβ42 monomers and oligomers. We further verified that bFg completely suppresses Aβ42-reduced cytotoxicity. To determine whether these bFg capabilities to suppress amyloid fibril formation and cytotoxicity depends solely on bFg tertiary structural features, we also examined hFg, which shares high structural similarity and moderate sequence similarity with bFg (Figure S1: Aα chain, 60%; Bβ chain, 82%; γ chain, 82%). hFg does not inhibit amyloid fibril formation or reduce cell toxicity as strongly as bFg does. Furthermore, hFg interacts only with the Aβ42 oligomer, and its interaction is weaker than that of bFg. Based on these findings, we discuss the molecular mechanisms underlying the inhibitory effects of fibrinogen molecules on Aβ42.

## Results

### Inhibition of Aβ42 Amyloid Fibril Formation by bFg and hFg

In this study, we first investigated if fibrinogen molecules inhibit Aβ42 amyloid fibril formation using a Thioflavin T (ThT) fluorescent probe. ThT is known to bind side chain grooves of amyloid fibrils in a perpendicular manner to the fibril axis.^21^ Figure 1A shows the time course of Aβ42 amyloid fibril formation at a concentration of 20 μM (0.09 mg/mL) at 37 °C, monitored by ThT fluorescence. The left and right panels represent the results obtained in the presence of bFg and hFg, respectively. In the absence of fibrinogen (black lines in each panel of Figure 1A), ThT intensity remained low for the first hour. At this stage, granular particles with a mean height of 5.0 ± 0.9 nm (*n* = 10) were observed (Figure 1B) and identified as oligomers. After 1 h of incubation, ThT intensity increased, suggesting the formation of amyloid fibrils. Figure 1C shows an AFM image obtained after 7 h, when the ThT intensity reached a plateau. Various fibril-like structures were observed, confirming the formation of amyloid fibrils. The sigmoidal increase in ThT intensity was analyzed using eq 1 to determine the half-time of the increase (τ_half_) and the intensity at the plateau phase (*I*_plat_). These values reflect the time constant required for amyloid fibril formation and the quantity of amyloid fibrils formed, and can thus be used to quantify the fibril formation curve. Although ThT intensities reflect the averages of amyloid-fibril polymorphs, their increase still serves as an indicator for the onset of fibril formation. For Aβ alone, τ_half_ = 2.0 ± 0.3 h, and *I*_plat_ = 0.66 ± 0.03 (Figure 1D).

**Figure 1:**
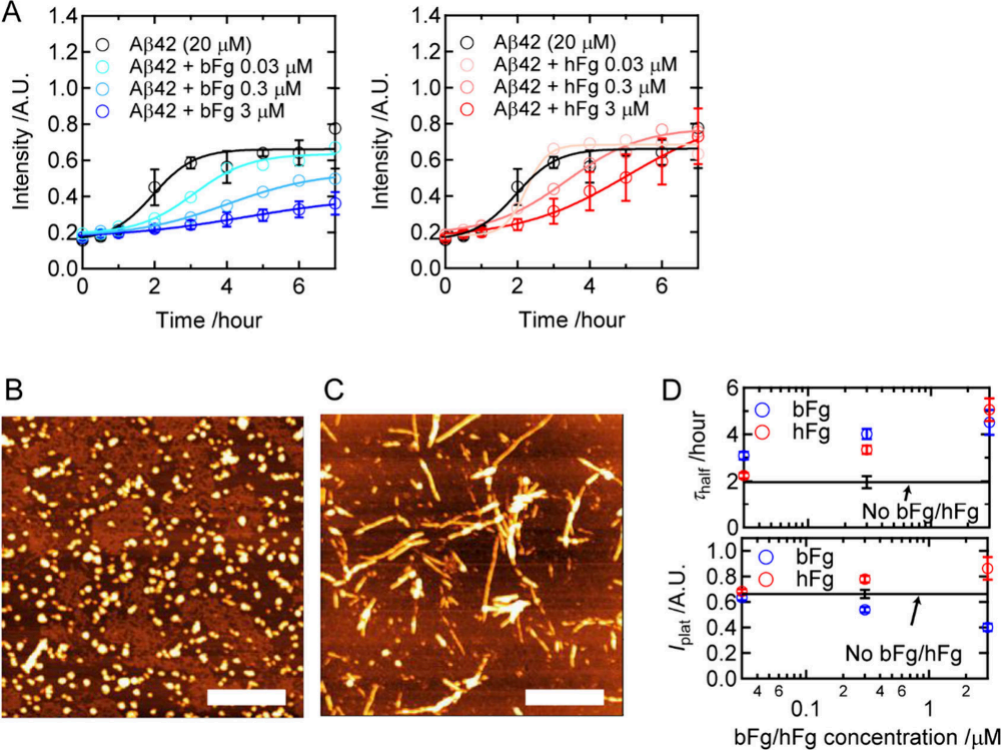
Effect of bFg and hFg on the amyloid fibril formation of Aβ42. (A) Time courses of ThT fluorescence intensities with bFg (left) or hFg (right). Error bars represent one standard deviation (*n* = 3). The lines indicate curves obtained by fitting using eq 1. (B) AFM image of an oligomer taken 1 h after incubation without bFg or hFg. Scale bar: 500 nm. (C) AFM image of amyloid fibrils taken 7 h after incubation without bFg or hFg. Scale bar: 500 nm. (D) The midpoint of the sigmoidal increase (τ_half_) and the fluorescence intensity at the plateau phase (*I*_plat_) obtained from curve fitting of the data in panel A using eq 1. Error bars represent values obtained from curve fitting. The value for the control (without bFg or hFg) is indicated by black lines.

To assess the inhibitory effects of bFg and hFg on Aβ amyloid fibril formation, Aβ was incubated with varying concentrations of bFg and hFg. As the bFg concentration increased, τ_half_ became longer, and *I*_plat_ decreased (Figure 1D), indicating that bFg inhibited fibril formation in a concentration-dependent manner. In the presence of hFg, τ_half_ increased only at 0.3 and 3 μM, and the effect at 0.3 μM was weaker compared to that of bFg. No decrease in *I*_plat_ was observed at any tested hFg concentration. These results represent that bFg exhibits a stronger inhibitory effect on amyloid fibril formation than hFg. It may be possible that aggregates of fibrinogen molecules contribute to the increase in the ThT intensity. To check the aggregate states of bFg and hFg, SEC-MALS, by which molecular distributions and molecular weights can be evaluated, was performed. As shown in Figure S2, both bFg and hFg were characterized by a single peak whose molecular weight was close to that of the monomer (∼340 kDa). This result suggests that both bFg and hFg were monomeric, and did not therefore contribute to the ThT intensity.

### Evaluating the Interaction between Aβ42 and Fibrinogen Molecules Using the Dot Blot Assay

Understanding the molecular mechanisms underlying amyloid fibril formation inhibition requires monitoring the interaction between Aβ42 and bFg or hFg at an early stage, before amyloid fibril formation occurs. For this purpose, sample solutions were collected 1 h after incubation began at 37 °C, a condition under which no apparent amyloid fibril formation was observed (Figure 1A), and the dot blot assay was performed using the solutions (Figure 2).

**Figure 2:**
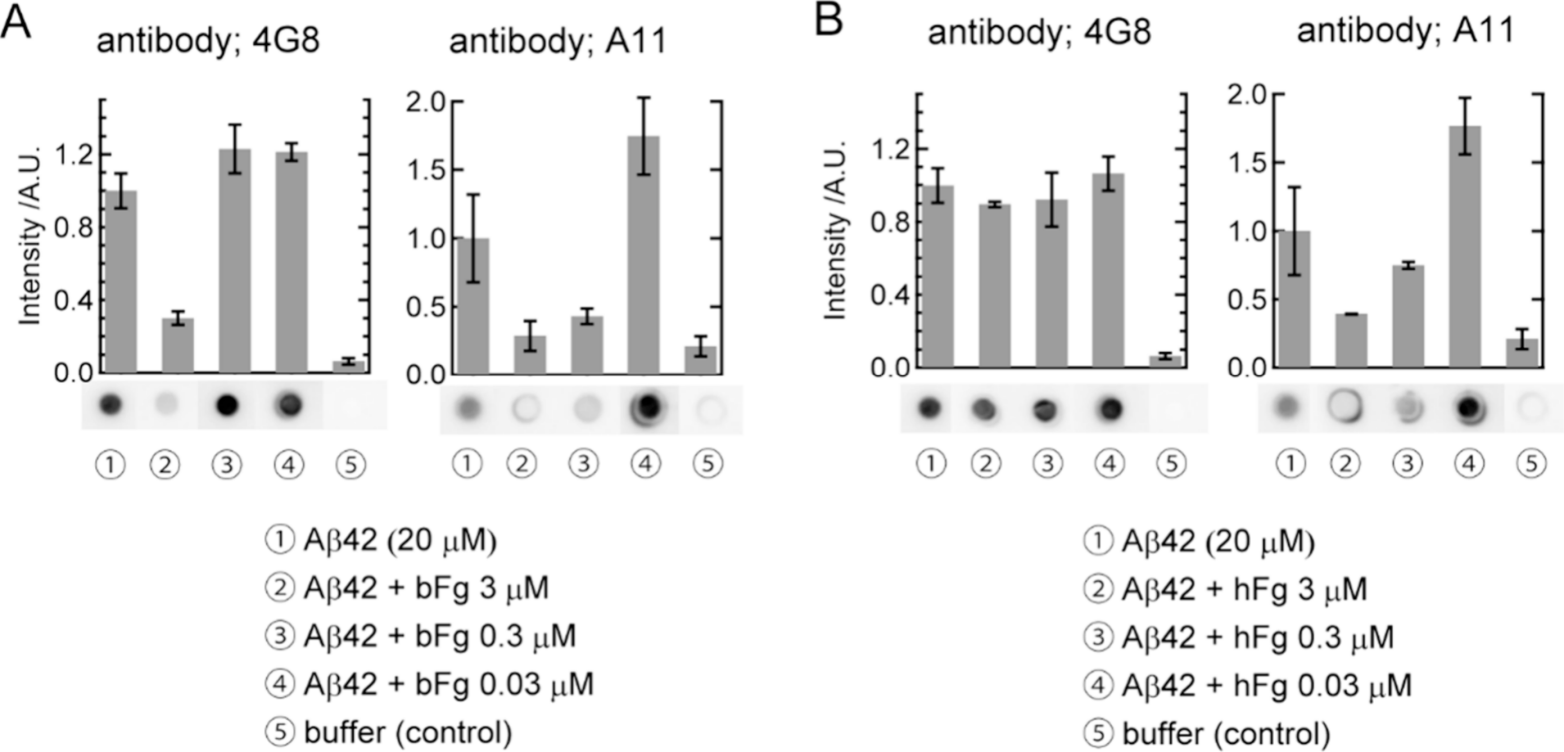
Dot blot results in the presence of bFg (A) or hFg (B), respectively. Samples were taken at 1 h after the start of amyloid fibril formation. Sample names are numbered and listed at the bottom of each panel. The intensities are normalized using that of Aβ42 only (sample ①). The error bars indicate one sigma values (*n* = 3 ∼ 15). Examples of blots are shown below the bars.

The mechanism of the dot blot assay is briefly explained here. If bFg or hFg interacts with Aβ42 monomers or oligomers, antibodies cannot bind to the epitope sequences of Aβ42 due to steric hindrance. Two antibodies, 4G8 and A11, were used in this experiment. 4G8, which recognizes an epitope at residues 17–24, interacts with Aβ42 monomers and oligomers^22,23^ and can therefore be used to evaluate the total amount of Aβ42 peptides at an early stage, before amyloid fibril formation. However, as shown in the AUC experiment performed under the same conditions as the dot blot assay, Aβ42 oligomer constituted approximately 10% of the sample population (Figure 3). Due to this low oligomer weight fraction, the contribution to the dot blot intensity would be at most around 10%. This value would be much smaller than that of monomers, which would account for 90% of the total dot blot intensity. Therefore, the intensity obtained with 4G8 is approximately proportional to the amount of monomers, serving as an indicator of monomer quantity. In contrast, A11 specifically detects Aβ42 oligomers by recognizing unique oligomeric structures, as reported in previous studies, and thus can be used as an indicator of oligomer quantity.^24,25^

**Figure 3:**
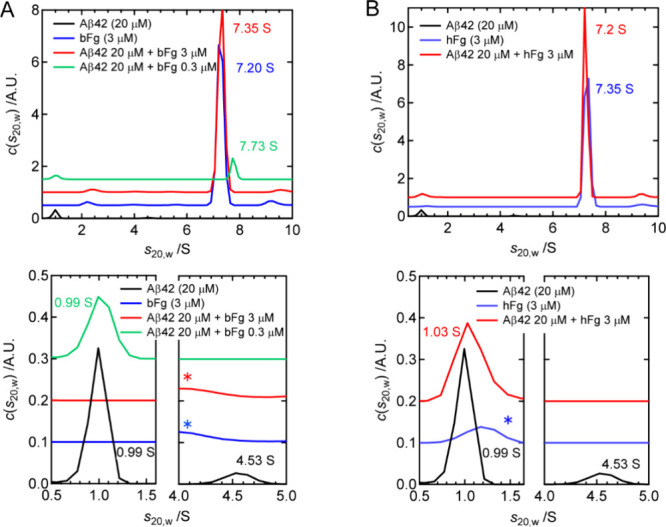
AUC profiles of Aβ42 in the presence of (A) bFg or (B) hFg at 1 h during amyloid fibril formation. Each panel displays an overview of the profiles (top) and magnified views along each axis (bottom). Each profile is shifted along the *y*-axis for clarity. Peaks marked with asterisks represent minor small components present in bFg or hFg.

Figure 2A shows the dot blot assay results when bFg was incubated with Aβ42. Compared with Aβ42 alone, the presence of bFg at 3 μM resulted in a marked reduction in spot intensity for both 4G8 and A11. At 0.3 μM bFg, a decrease was observed only with A11, while at 0.03 μM bFg, no significant decrease was observed with either antibody. These findings indicate that bFg interacts with both Aβ42 monomers and oligomers at 3 μM but interacts only with oligomers at 0.3 μM. Figure 2B shows the dot blot assay results following incubation with hFg. At an hFg concentration of 3 μM, spot intensity decreased for A11 but not for 4G8. No significant decrease in spot intensity for A11 was observed at lower hFg concentrations. This suggests that hFg interacts only with Aβ42 oligomers at 3 μM and that this interaction is weaker than that of bFg, as the interaction of oligomers with bFg was still detectable at 0.3 μM. Notably, when A11 was used, the spot intensities were higher in the presence of 0.03 μM bFg or hFg than in samples without fibrinogen molecules (right panels of Figures 2A and 2B), though the reason is unclear. To exclude the possibility that 4G8 and A11 cross-react with bFg and hFg, we performed dot blot assay of bFg and hFg in the absence of Aβ as a control. As shown in Figure S3, the spot intensities of bFg and hFg were as low as those of buffer for both 4G8 and A11, suggesting that bFg or hFg does not interact with the antibodies and thus guaranteeing the antibodies’s specificities.

### Evaluating the Interaction between Aβ42 and Fibrinogen Molecules by AUC

AUC was performed to investigate the molecular interactions between Aβ42 and Fg at the same time point as the dot blot assay (1 h after sample incubation at 37 °C). Figure 3A shows the AUC profiles of Aβ42, bFg, and their mixtures. For Aβ42, two peaks were observed at 0.99 and 4.53 S, corresponding to monomeric Aβ42 and its oligomer, respectively. Based on the peak areas, the monomer population was estimated to be 90%, while that of the oligomer was 10%. Both components disappeared in the presence of bFg at a concentration of 3 μM, and only a peak at 7.35 S, corresponding to bFg, was observed. The Aβ42-bFg complex may also be contained in this peak. When the bFg concentration was reduced to 0.3 μM, the oligomer peak disappeared, whereas the monomer peak remained. These results are consistent with those of the dot blot assay showing that the spot intensity of A11 significantly reduced whereas that of 4G8 unchanged (Figure 2A). Figure 3B presents the AUC profiles of Aβ42 mixed with hFg. In the presence of hFg at a concentration of 3 μM, the Aβ42 oligomer component disappeared, whereas the monomer peak remained, consistent with the results of dot blot assay showing that the spot intensity of A11 largely decreased while that of 4G8 unchanged (Figure 2B).

Overall, when a significant decrease in the spot intensity in dot blot was observed in the presence of bFg or hFg (Figure 2), peak disappearance of the Aβ42 monomers or oligomers occurred due to the interaction with the fibrinogen molecules (Figure 3). This correlation is further supported when AUC intensities are plotted against dot blot intensities (Figure S4). The complete peak disappearance in AUC may reflect the amount of residual free Aβ species falling below the detection limit of the method.

### Confirmation of Fibrinogen Molecule Interaction with Aβ42 Oligomers by AFM

Figure 4 presents AFM images of Aβ42 in the absence and presence of bFg or hFg, captured at the same time point as the dot blot and AUC experiments (1 h after incubation at 37 °C). The Aβ42 and fibrinogen concentrations were 20 μM and 3 μM, respectively. A large number of oligomers were observed in the absence of fibrinogen molecules (Figure 4A). However, these oligomers were rarely observed in the presence of bFg or hFg (Figure 4B and 4C), suggesting that fibrinogen molecules inhibited the interaction of the oligomers with the mica surface. Our previous study showed that bFg was not detectable using the same AFM method due to weak interaction with the mica surface.^19^ Based on this fact, the present result implies that bFg interacted with the oligomer surface, shielding the interaction of oligomers with the mica surface. A similar molecular mechanism may apply to hFg.

**Figure 4:**
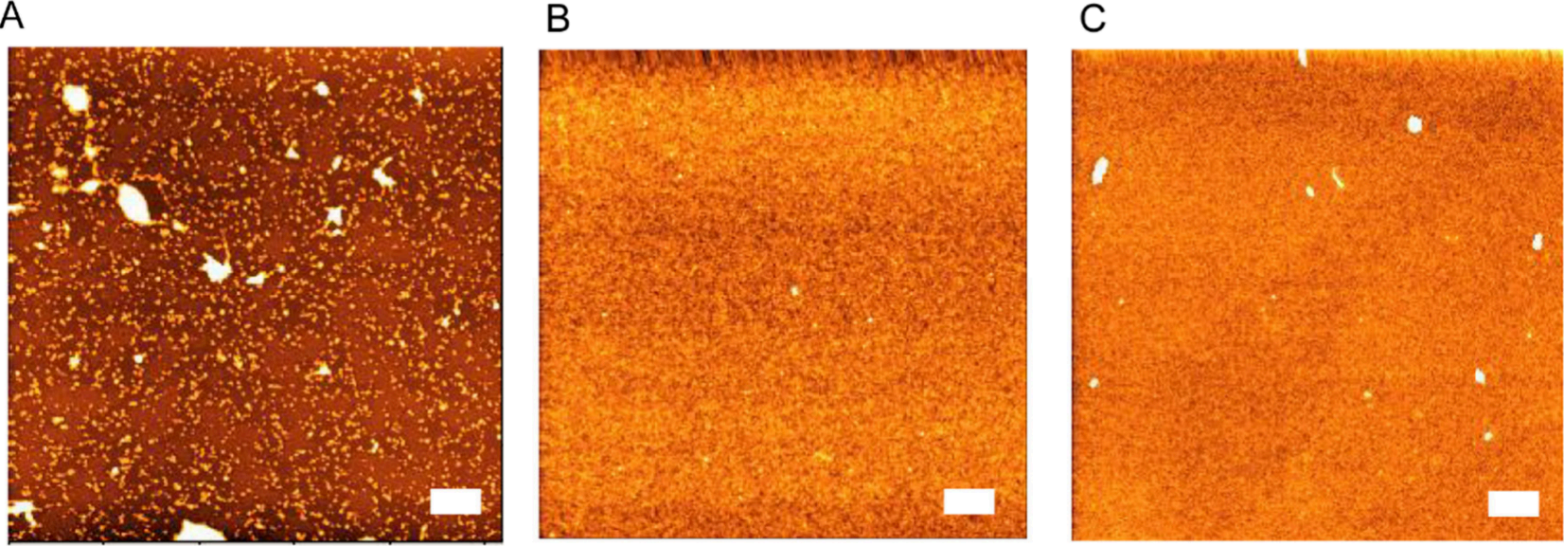
Effect of bFg and hFg on Aβ42 oligomer formation, as observed in AFM images taken 1 h after the start of amyloid fibril formation. (A) Aβ42 only. (B) With 3 μM bFg. (C) With 3 μM hFg. Scale bars indicate 500 nm.

### Inhibition Effect of bFg and hFg on Aβ42-Induced Cytotoxicity

We evaluated the cytotoxicity of Aβ42 in the absence and presence of bFg or hFg using an MTT assay. Aβ42 at 20 μM, with or without fibrinogen molecules at 3 μM, was incubated under the same conditions as in the dot blot and AUC experiments (1 h at 37 °C) before being added to the cell culture. Figure 5 presents the cell viability results obtained from the MTT assay. The cell viability decreased to approximately 60% following incubation with Aβ42 alone. In the presence of bFg, cell viability recovered to nearly 100%, whereas hFg led to only approximately 80% recovery. These results indicate that bFg fully suppresses Aβ42-induced cytotoxicity, whereas hFg only partially suppresses it.

**Figure 5:**
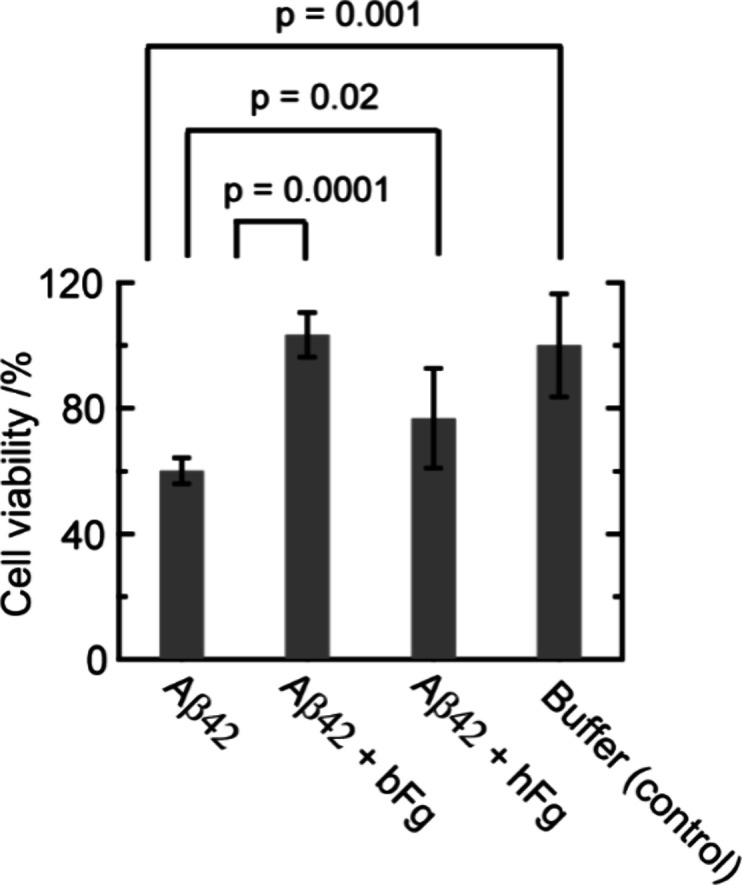
Cell viability in the presence of Aβ42 with or without bFg or hFg. Error bars represent ± 2 SD (*n* = 3). P-values were obtained by a one-sided two-sample *t* test assuming equal variances.

## Discussion

### Molecular Mechanisms Underlying the Interaction between Aβ42 and Fibrinogen Molecules

Based on the findings of this study, the molecular mechanisms underlying the interaction between Aβ42 and fibrinogen molecules are discussed (Figure 6). We found that bFg binds to both Aβ42 monomers and oligomers, whereas hFg interacts only with oligomers and more weakly than bFg (Figures 2, 3, and 4). Here, we calculate the number of oligomer-binding sites on fibrinogen molecules. Assuming that oligomer particles are spherical with a diameter of 5 nm (Figure 1B) and considering the molecular weight of Aβ42 as 4512 g/mol and the mean protein density as 1.35 g/cm^3^,^26,27^ each sphere would contain 12 monomers. This suggests that the average oligomer consists of 12 monomers, which is consistent with previously reported oligomers.^28^ Assuming that the oligomer is predominantly a 12mer, its effective concentration would be approximately 0.17 μM (calculated as 20 μM × 0.1/12, given that oligomers constitute 10% of the total Aβ42 and are composed of 12 monomers). Because bFg at 3 μM interacted with Aβ42 oligomers, and this interaction persisted at 0.3 μM of bFg (Figure 2 and 3), at least approximately two binding sites in bFg are required to sustain interaction at 0.3 μM (Figure 2 and 3). Since bFg consists of dimeric subunits, one binding site is likely present on each subunit (Figure 6, binding manner of the oligomer). hFg might also share the same binding site for Aβ oligomers as bFg. However, hFg at 0.3 μM did not exhibit binding to oligomers (Figure 2), indicating that the interaction between bFg and oligomers is stronger than that between hFg and oligomers. To quantify the binding affinities, apparent *K*_d_ values (*K*_d_^app^) were obtained by analyzing dot blot results based on a binding scheme, eq S1, described in Supporting Information. As shown in Figure S5 A and B, the dot blot results were well fitted by eq S6, obtaining *K*_d_^app^ values of 0.20 ± 0.05 μM and 1.4 ± 0.2 μM for bFg and hFg, respectively. The smaller *K*_d_^app^ for bFg than that for hFg is consistent with the stronger interaction of bFg to Aβ oligomers than hFg (Figure 6, “*Stronger binding*” between bFg and hFg).

**Figure 6:**
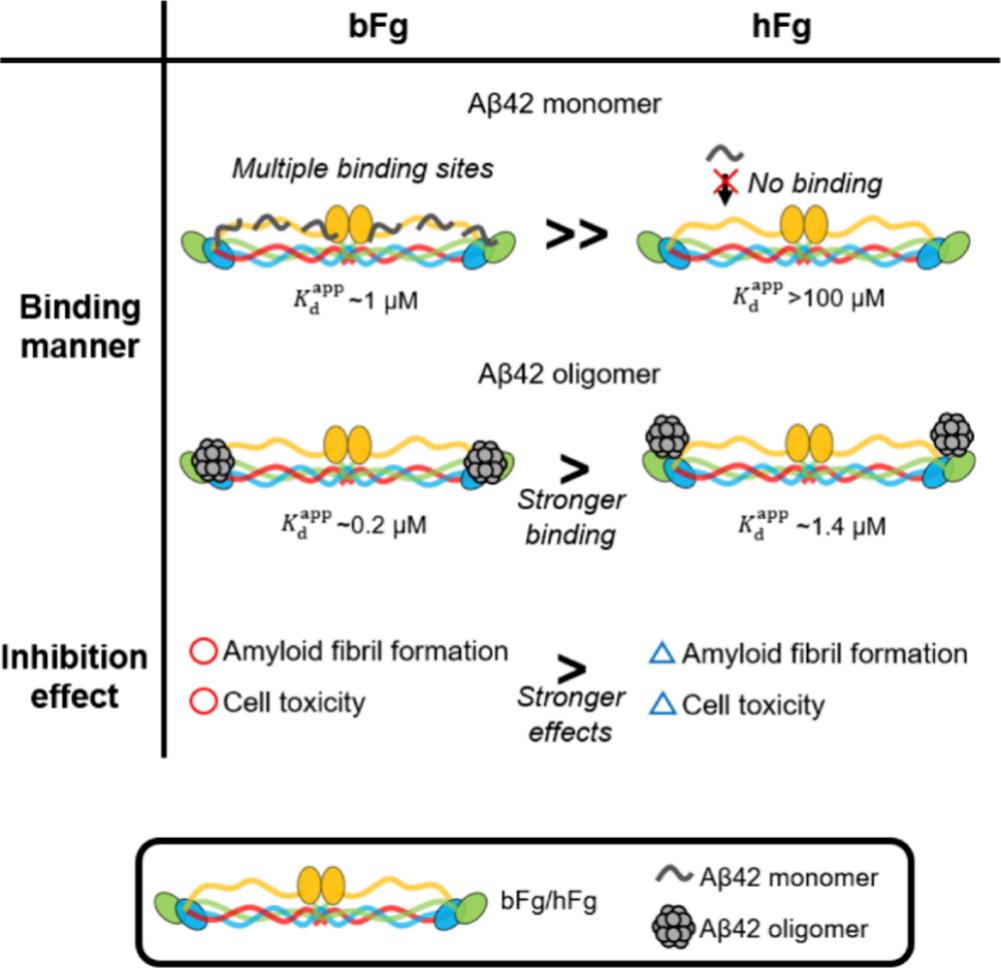
Proposed binding manners between Aβ42 and the fibrinogen molecules, and resultant effects on the inhibition of the Aβ42 amyloid fibril formation and cell toxicity. *K*_d_^app^ values were obtained using eq S6. The representations of the colors of bFg or hFg molecules are as follows: The N-terminal region of Aα chain, red; the C-terminal region of Aα chain, i.e., αC region, orange; the Bβ chain, blue; the γ chain, green. Globular domains are represented by ellipses. See details in the main text.

bFg also interacted extensively with Aβ42 monomers at 3 μM (Figures 2 and 3). The concentration of Aβ42 monomers was estimated to be approximately 18 μM (calculated as 20 μM × 0.9, given that ∼ 90% remained in monomeric form, as revealed by AUC in Figure 3). Based on this, at least six binding sites would be required for bFg to interact with Aβ42 monomers (Figure 6, “*Multiple binding sites*” at bFg). In contrast, hFg did not interact with monomers within the examined concentration range (Figure 2 and 3), implying much smaller affinity with monomers than bFg (Figure 6, “*No binding*” at hFg). To estimate the binding affinities, the dot blot results were simulated using eq S6. As shown in Figure S5 C and D, *K*_d_^app^ for bFg was at the order of 1 μM whereas that for hFg was at the order of 100 μM or more, consistent with the fact that only bFg binds to Aβ monomers (Figure 6).

At this stage, the specific binding sites for Aβ42 on bFg and hFg remain unknown. However, several potential sites have been suggested in the literature. Aβ42 has been reported to interact with hFg at two regions: one located between amino acid residues 240 and 421 in the Aα chain and another between residues 366 and 414 in the Bβ chain.^29,30^ These sites may interact with Aβ42 oligomers. Given that bFg shares homology with hFg, the corresponding sites in bFg could also serve as binding regions for Aβ42 oligomers. However, considering the presence of six binding sites of bFg for Aβ42 monomers, additional binding regions must exist in bFg beyond those involved in oligomer binding. Sequence identity between bFg and hFg is high for the Bβ (82%) and γ (82%) chains but relatively low for the Aα chain (60%), as determined using CLUSTAL W^31^ (Figure S1). Notably, the C-terminal αC region in the Aα chain (residues 221–610 of the Aα chain in hFg and the corresponding region in bFg) exhibits substantial sequence divergence between bFg and hFg (52% sequence similarity, Figure S1). This region is known to be relatively flexible and can engage in intermolecular interactions to facilitate clot formation upon thrombin digestion.^16^ This sequence difference in the αC region may contribute to the differential binding affinities of bFg and hFg for Aβ42 (Figure 6, Aβ42 monomer binding on the αC region). Further structural studies are required to clarify these binding interactions in greater detail.

### Inhibition Mechanism of the Amyloid Fibril Formation

Amyloid fibrils generally form from monomeric units through two primary pathways.^32,33^ One pathway involves the direct nucleation of monomers into amyloid fibrils, known as the nucleation–growth (NG) model. The second pathway involves amyloid fibril formation from oligomers or protofibrils that arise before fibril formation, referred to as the nucleus conformational conversion (NCC) model. In realistic conditions, i.e. *in vivo*, both models may coexist in varying proportions.^34^ In the NG model, the interaction of fibrinogen molecules with Aβ42 monomers plays a crucial role in inhibiting amyloid fibril formation. Conversely, in the NCC model, interactions with both Aβ42 monomers and oligomers can suppress fibril formation. Therefore, bFg, which binds to both Aβ42 monomers and oligomers (Figure 2 and 3) with *K*_d_^app^ values on the order of μM and sub-μM ranges (Figure S5 C and A), could suppress fibril formation by preventing both the NG and NCC pathways. In contrast, hFg, which binds only to oligomers (Figure 2 and 3), may inhibit fibril formation mainly by interfering with the NCC pathway. Furthermore, because hFg interacts with oligomers more weakly than bFg does as shown by the difference in their *K*_d_^app^ values (Figure S5 A and B), its inhibitory effect via the NCC pathway may be weaker. This difference in both the interaction specificity and strength between bFg and hFg may explain the greater inhibitory capability of bFg in fibril formation (Figure 6, “*Stronger effects*” of bFg than hFg). Further validation should be performed to clarify the molecular mechanisms of inhibition in more detail.

### Inhibition Mechanism of Cell Toxicity by bFg or hFg

Both Aβ42 monomers and oligomers are known to induce cell toxicity by interacting with cell membranes.^6,7^ Therefore, binding of bFg or hFg to these species is essential for suppressing toxicity. Indeed, bFg at 3 μM, which binds both Aβ42 monomers and oligomers (Figure 2 and 3), completely suppressed cell toxicity (Figure 5). In contrast, hFg at 3 μM, which binds only to oligomers (Figure 2 and 3), exhibited only partial inhibition of cell toxicity (Figure 5). These results strongly indicate that the ability of bFg to bind both monomers and oligomers is critical for suppressing cell toxicity (Figure 6, “Stronger effects” of bFg than hFg). Furthermore, amyloid fibrils themselves are also known to be cytotoxic.^35^ Because bFg exhibits a greater ability to suppress amyloid fibril formation than hFg (Figure 1A), it is possible that this suppression further contributes to the reduction of Aβ42 cytotoxicity. Under realistic conditions, both binding to Aβ42 monomers and oligomers and the consequent suppression of amyloid fibril formation likely contribute to the inhibition of cell toxicity.

### Implications for the Clinical Use of bFg

Various chaperone proteins such as Hsp104 ^36^ and chaperone-like proteins such as α2-macroglobulin, haptoglobin,^37^ and human serum albumin,^38^ are known to inhibit Aβ42 amyloid fibril formation. However, these proteins primarily target intermediate species, such as oligomers or protofibrils, rather than monomers. In contrast, bFg, which binds to both Aβ42 oligomers and monomers, has the potential to suppress amyloid fibril formation at earlier stages than these proteins. Thus, bFg represents a promising candidate for therapy of Alzheimer’s disease.

Conversely, Aβ42 interaction with hFg has been shown to induce its aggregation, leading to the formation of more stable fibrin clots via thrombin-mediated reactions. Such clots are more resistant to plasmin-mediated degradation, which can promote inflammation.^29^ Furthermore, Aβ42 and fibrin coaccumulate in cerebral vessels in patients with Alzheimer’s disease, contributing to cerebral amyloid angiopathy,^39^ though Aβ40 is the predominant isoform.^40^ Given that bFg is a homologue of hFg, its potential for similar clot formation necessitates careful consideration in its therapeutic application. Despite these concerns, bFg remains a promising candidate for Alzheimer’s disease treatment. One possible approach is to remove bFg at an appropriate time after its introduction into the brain, for example, using bFg-specific antibodies. Another approach could involve coadministration of bovine plasmin to prevent undesirable clot formation with Aβ42. Further fundamental and clinical studies are necessary to explore and optimize the therapeutic potential of bFg for Alzheimer’s disease.

## Conclusions

In this study, using various methods such as dot blot assay, AUC, and AFM, we demonstrated that bFg inhibits amyloid fibril formation by binding to both the monomer and oligomer of Aβ42. In contrast, hFg, which binds only to the oligomer, exhibited a weaker inhibitory effect than bFg. Furthermore, bFg completely suppressed cell toxicity of Aβ42, whereas hFg only partially inhibited it. These findings indicate that interaction with both the monomer and oligomer, which are present in the early stage preceding amyloid fibril formation, is essential for effectively suppressing amyloid fibril formation and Aβ42-induced cell toxicity. Therefore, molecules capable of binding both the monomer and oligomer of Aβ42 may represent promising therapeutic candidates for Alzheimer’s disease. Our *in vitro* study may thus help design further therapeutic strategies focusing on the early molecular species.

## Materials and Methods

### Proteins and Peptides

Human and bovine fibrinogens (hFg and bFg, respectively) from bovine plasma (Wako Pure Chemical Industries, Osaka, Japan) were purified via size-exclusion chromatography. Briefly, approximately 50 mg of powder was dissolved in 1 mL of buffer solution (100 mM Tris-HCl and 10 mM HCl, pH 8.5, or 100 mM phosphate and 400 mM NaCl, pH 7.4) and purified using a HiPrep 16/60 Sephacryl S-300 HP column (GE Healthcare, NY) equipped with an ÄKTA Start system (GE Healthcare, NY). Monomer fractions were collected and stored at −80 °C. If necessary, the buffer component was replaced with those used for other experiments using an Amicon Ultra-15 30 kDa centrifugal filter (Merck Millipore, Germany).

Amyloid beta peptide (Aβ42) was purchased from Peptide Institute, Inc. (Japan) and used without further purification. Approximately 0.5 mg of powder was dissolved in 500 μL of 0.05% NH_3_ solution, and the concentration was determined using an extinction coefficient of ε = 1,411 M^–1^ cm^–1^ at 280 nm. The solution was stored at −80 °C.

### Amyloid Fibril Formation

The stock Aβ42 solution in 0.05% NH_3_was diluted to a concentration of 143 μM. A 28-μL aliquot of the diluted solution was mixed with 63 μL of Milli-Q water, 9 μL of 100 mM HCl solution, and 100 μL of 100 mM phosphate/400 mM NaCl solution (pH 7.4) to obtain a final concentration of 20 μM Aβ42 in 50 mM phosphate/200 mM NaCl (pH 7.4). When bFg or hFg was incubated with Aβ42, 100 μL of bFg or hFg solution at an arbitrary concentration in 100 mM phosphate/400 mM NaCl solution (pH 7.4) was used instead of the 100 mM phosphate/400 mM NaCl solution. The final mixture was incubated at 37 °C for fibril formation.

### Thioflavin T (ThT) Fluorescence Assay

Amyloid fibril formation was monitored using ThT fluorescence. For the assay, a 5-μL sample was mixed with 1 mL of ThT solution (5 μM ThT in 50 mM Gly-NaOH, pH 8.5), and the fluorescence intensity at 485 nm was measured with excitation at 445 nm using an FP-6500 spectrofluorometer (JASCO, Japan). The time course of ThT intensity was analyzed using the following equation:

$$\mathrm{ThT intensity}\left(t\right)=I_{\mathrm{base}}+\left[\frac{I_{\mathrm{sigm}}}{1+\exp\left\{\frac{\tau_{\mathrm{half}}-t}{\tau_{\mathrm{rate}}}\right\}}\right]$$ 1

where *I*_base_ represents the baseline intensity, which was fixed at the value at 0 h. *I*_sigm_ indicates the increase in intensity of the sigmoidal curve. τ_half_ denotes the time at which the sigmoidal increase reaches half of the final value, and τ_rate_ defines the slope of the sigmoidal increase. The intensity at the plateau phase, *I*_plat_, was defined as *I*_base_+*I*_sigm_.

### AFM

A 5-μL sample was loaded onto a mica plate, incubated for 1 min, and rinsed with 1 mL of Milli-Q water. Residual moisture naturally dried. AFM images were captured in dynamic force mode using a Probestation NanoNavi II/IIe (SII Nanotechnology). The force set point was automatically optomized for each sample. An OMCL-AC160TS-C3 cantilever (OLYMPUS) was used. The sweep rate was set to 0.5 Hz, and images were recorded with 256 × 256 points per image.

### Dot Blot Assay

Dot blotting was performed using a Bio-Dot system (Bio-Rad Laboratories, Inc., Boston, MA, USA) according to the manufacturer’s instructions. Each assay was conducted with a 10-μL sample. Two primary antibodies, 4G8 (BioLegend, San Diego, CA) and A11 (StressMarq Biosciences Inc., Canada), were used to detect monomeric and oligomeric Aβ42, respectively. Horseradish peroxidase (HRP)-conjugated horse antimouse IgG (Cell Signaling Technology, Danvers, MA) and HRP-conjugated goat antirabbit IgG (LGC Clinical Diagnostics, Milford, MA) were used for 4G8 and A11, respectively. All antibodies were diluted 1:2000 in TBS-T, and 100 μL of antibody solution was used per assay. Blot spots were captured using an AMERSHAM ImageQuant 800 system (GE Healthcare, NY), and analyses were performed using ImageQuant TL Version 8.2 (GE Healthcare, NY, USA).

### AUC

AUC measurements were performed using a ProteomeLab XL-I analytical ultracentrifuge (Beckman Coulter, Germany) equipped with an aluminum cell with a volume of 400 μL (optical path: 12 mm). Sedimentation velocity AUC was determined using Rayleigh interference optics. Samples were prepared as described in the amyloid fibril formation section and loaded into the cells. Samples were incubated in the rotor, pre-equilibrated at 37 °C, for 1 h before initiating rotation at 37 °C. The rotor speed was set to 40,000 rpm for all experiments. The weight-concentration distribution, *c*(*s*_20,w_), as a function of sedimentation coefficient, was obtained using SEDFIT software version 15.01c (https://sedfitsedphat.github.io/). The fitting analysis was performed based on the Lamm equation.^41^ The sedimentation coefficient was converted to its value at 20 °C in pure water,*s*_20,w_.

### Cell Viability Assay

Cell viability assays were performed using the MTT Cell Count Kit (Nacalai Tesque, Kyoto, Japan). Jurkat cells were obtained from the Health Science Research Resources Bank (Osaka, Japan) and cultured in RPMI 1640 medium (Sigma-Aldrich, St. Louis, MO). Aβ42 samples, with or without fibrinogen molecules, were prepared as described in the amyloid fibril formation section. The samples were incubated at 37 °C for 1 h, after which 5 μL of each sample was mixed with 95 μL of the cell suspension. The mixture was then incubated at 37 °C with 5% CO_2_ for 24 h. Cell viability was assessed using MTT reagent, and absorbance at 570 nm was measured using the GloMax Explorer Multimode Microplate Reader (Promega, Madison, WI).

## Supplementary Material



## Notes

SI methods, sequence alignments between bFg and hFg for Aα chain, Bβ chain, and γ chain (Figure S1), SEC-MALS results of bFg and hFg (Figure S2), dot blot result of bFg and hFg (Figure S3), correlations between dot blot and AUC intensities for Aβ monomers and oligomers (Figure S4), analysis of dot blot result for A11 and 4G8 (Figure S5) (PDF)
