# Supplementary material for: Targeting Both Monomer and Oligomer with Fibrinogen Efficiently Suppresses Amyloid Fibril Formation and Cell Toxicity of Amyloid β 1‑42
Source: ACS Chem Neurosci. 2025 Sep 5;16(18):3623–30. doi: 10.1021/acschemneuro.5c00562 (PMC12447488; doi:10.1021/acschemneuro.5c00562)
Supplement: Supplementary file 1 [file cn5c00562_si_001.pdf]

Supporting Information (SI)

Targeting both monomer and oligomer with fibrinogen efficiently suppresses amyloid fibril formation and cell toxicity of amyloid β 1-42

Authors: Naoki Yamamoto[a]*, Keisuke Yuzu[b], Ken Morishima[c], Rintaro Inoue[c], Masaaki Sugiyama[c], Daisuke Koyama[d], and Eri Chatani[b]

Affiliation

[a] Division of Biophysics, Physiology, School of Medicine, Jichi Medical University, 3311-1 Yakushiji, Shimotsuke, Tochigi, 329-0498, Japan

[b] Graduate School of Science, Kobe University, 1-1 Rokkodai-cho, Nada-ku, Kobe, 657-8501, Japan

[c] Institute for Integrated Radiation and Nuclear Science, Kyoto University, 2 Asashiro-Nishi, Kumatori, Sennan-gun, Osaka, 590-0494, Japan

[d] Department of Hematology, Fukushima Medical University, Fukushima, 960-1295, Japan

*Corresponding author

Tel.: +81-285-58-7308, Fax.: +81-285-40-6294, E-mail: nyamamoto@jichi.ac.jp

SI methods

SEC-MALS

A sample of 190 μL was applied to a Superose 6 Increase 10/300 column (Cytiva, NY) equipped on an ÄKTA purifier (GE Healthcare, NY). The elution was performed at 0.5 mL/min at 4 °C using the same buffer as the sample. The eluate was analyzed using a DAWN 8 MALS detector (Wyatt Technology, CA) and a UV-4575 detector (JASCO, Japan). The MALS data were analyzed using ASTRA software (Wyatt Technology, CA) to calculate the molecular weight.

Analysis of dot blot assay

Dot blot data were analyzed using a biomolecular interaction model (Heymann et al., *Biochemistry* 1996, 35, 2717-2725). In this model, one Aβ molecule interacts with a binding site consisting of *n* fibrinogen molecules (Fg). The reaction scheme is represented as

$$\mathrm{A\beta} + \mathrm{Fg}_n \leftrightarrows \mathrm{A\beta\text{-}Fg}_n \qquad \text{–(S1)}$$

where Aβ, Fg_n, and $\mathrm{A\beta\text{-}Fg}_n$ represent the free Aβ species, *n* free Fg molecules, and the complex of Aβ with *n* Fg molecules, respectively. The apparent dissociation constant, $K_\mathrm{d}^\mathrm{app}$, for the interaction between one Aβ species and one binding unit composed of *n* Fg molecules is defined as

$$K_\mathrm{d}^\mathrm{app} = \frac{[\mathrm{A\beta_f}][\mathrm{Fg_f}]}{n[\mathrm{A\beta_b}]} \qquad -\text{(S2)}$$

where $[\mathrm{A\beta_f}]$ and $[\mathrm{A\beta_b}]$ are the concentrations of the free and bound Aβ species, respectively, and $[\mathrm{Fg_f}]$ is the concentration of the free Fg molecules. The concentration of Fg molecules bound to Aβ is given by $[\mathrm{Fg_b}] = \mathrm{n}[\mathrm{A\beta_b}]$. The total fibrinogen concentration, [Fg], and the total Aβ concentration, [Aβ], are given by:

$$[\mathrm{A\beta}] = [\mathrm{A\beta_f}] + [\mathrm{A\beta_b}] \qquad \text{-(S3)}$$

$$[\mathrm{Fg}] = [\mathrm{Fg_f}] + [\mathrm{Fg_b}] = [\mathrm{Fg_f}] + \mathrm{n}[\mathrm{A\beta_b}] \qquad \text{-(S4)}$$

Solving eqs. S2-S4 yields the fraction of the free Aβ species, f_f at equilibrium, given by

$$f_{\mathrm{f}} = \frac{1}{2}\left[1 - \frac{[\mathrm{Fg}]}{n[\mathrm{A\beta}]} - \frac{K_{\mathrm{d}}^{\mathrm{app}}}{[\mathrm{A\beta}]} + \sqrt{\left(1 - \frac{[\mathrm{Fg}]}{n[\mathrm{A\beta}]} - \frac{K_{\mathrm{d}}^{\mathrm{app}}}{[\mathrm{A\beta}]}\right)^2 + \frac{4K_{\mathrm{d}}^{\mathrm{app}}}{[\mathrm{A\beta}]}}\right]. \qquad -\text{(S5)}$$

The Fg concentration-dependent dot blot intensity, I([Fg]), for A11 was fitted using the equation

$$I([\mathrm{Fg}]) = (I_0 - I_\infty) f_{\mathrm{f}} + I_\infty \qquad \text{-(S6)}$$

where I_0 and I_∞ are the spot intensities of the Aβ-only sample and buffer, respectively, which were fixed in the fitting. A11 data were fitted to obtain $K_{\mathrm{d}}^{\mathrm{app}}$ for the interaction between Aβ oligomers and Fg molecules. In the fitting, n and [Aβ] were fixed at 0.5 and 0.17 μM, respectively. Data points at 0.03 μM bFg or hFg were excluded from the fitting. Simulation of 4G8 results was performed using eq. S6 to estimate $K_{\mathrm{d}}^{\mathrm{app}}$ for the interaction between Aβ monomers and Fg molecules by fixing n and [Aβ] at 0.17 (1/6) and 18 μM, respectively.

Figure S1

A

```
bFg Aα chain     EDGSDPPSGDFLTEGGGVRGPRLVERQQSACKETGWPFCSDEDWNTKCPSGCRMKGLIDE 60
hFg Aα chain     ---ADSGEGDFLAEGGGVRGPRVVERHQSACKDSDWPFCSDEDWNYKCPSGCRMKGLIDE 57
                    :*. .****:*********:***:*****::.********** **************

bFg Aα chain     VDQDFTSRINKLRDSLFNYQKNSKDSNTLTKNIVELMRGDFAKANNNDNTFKQISEDLRS 120
hFg Aα chain     VNQDFTNRINKLKNSLFEYQKNNKDSHSLTTNIMEILRGDFSSANNRDNTYNRVSEDLRS 117
                 *:****.*****::***:****.***::**.**:*::****:.***.***::::******

bFg Aα chain     RIEILRRKVIEQVQRIKVLQKNVRDQLVDMKRLEVDIDIKIRSCKGSCSRALEHKVDLED 180
hFg Aα chain     RIEVLKRKVIEKVQHIQLLQKNVRAQLVDMKRLEVDIDIKIRSCRGSCSRALAREVDLKD 177
                 ***:*:*****:**:*::****** *******************:******* ::***:*

bFg Aα chain     YKNQQKQLEQVIAINLLPSRDIQYLPLIKMSTITGPVPREFKSQLQEAPLEWKALLEMQQ 240
hFg Aα chain     YEDQQKQLEQVIAKDLLPSRDRQHLPLIKMKPVPDLVPGNFKSQLQKVPPEWKALTDMPQ 237
                 *::********** :****** *:******..:.. ** :******:.* ***** :* *

bFg Aα chain     TKMVLETFGGDGHARGDSVSQGTGLAPGSPRKPGTSSIGNVNPGSYGPGSSGTWNPGRPE 300
hFg Aα chain     MRMELERPGGNEITRGGSTSYGTGSETESPRNP--SSAGSWNSGSSGPGSTGNRNPGSSG 295
                  :* **  **:  :**.*.* ***  . ***:*  ** *. *.** ****:*. *** .

bFg Aα chain     PGSAGTWNPGRPEPGSAGTWNPGRPEPGSAGTWNPGRPEPGSAGTWNPGRPEPGSAGTWN 360
hFg Aα chain     TGGTATWKPGSSGPGSTGSWNSGSSGTGSTGNQNPGSPRPGSTGTWNPGSSERGSAGHWT 355
                 .*.:.**:** . ***:*:**.* . .**:*. *** *.***:****** .* **** *.

bFg Aα chain     TGSSGS----------SSFRPDSSGHGNIRPSSPDWGTFREE-GSVSSGTKQEFHTGKLV 409
hFg Aα chain     SESSVSGSTGQWHSESGSFRPDSPGSGNARPNNPDWGTFEEVSGNVSPGTRREYHTEKLV 415
                 : ** *          .******.* ** **..******.*  *.**.**::*:** ***

bFg Aα chain     TTKGDKELLIDNEKVTSGHTTTTRRSCSKVITKTVTNADGRTETTKEVVKSEDGSDCGDA 469
hFg Aα chain     TSKGDKELRTGKEKVTSGSTTTTRRSCSKTVTKTVIGPDGHKEVTKEVVTSEDGSDCPEA 475
                 *:******  .:****** **********.:**** ..**:.*.*****.******* :*

bFg Aα chain     DFDWHHTFPSRGNLDDFFHRDKDDFFTRSSHEFDGRTGLAPEFAALGESGSS-------- 521
hFg Aα chain     MDLGTLSGIGTLDGFRHRHPDEAAFFDTASTGKTFPGFFSPMLGEFVSETESRGSESGIF 535
                       :  .  :     * *:  **  :*        ::* :. : .. .*

bFg Aα chain     -----------------SSKTSTHSKQFVSSSTTVNRGGSAIESKHFKMEDEAESLEDLG 564
hFg Aα chain     TNTKESSSHHPGIAEFPSRGKSSSYSKQFTSSTSYNRGDSTFESKSYKMADEAG--SEAD 593
                                  *  .*:  .: .:***: ***.*::*** :** ***   .: .

bFg Aα chain     FKGAHGTQKGHTKARPARGIHTSPLGEPSLTP 596
hFg Aα chain     HEGTHSTKRGHAKSRPVRGIHTSPLGKPSLSP 625
                 .:*:*.*::**:*:**.*********:***:*
```

B

```
bFg Bβ chain     QFPTDYDEGQDDRPKVGLGARGHRPYDKKKEEAPSLRPVPPPISGGGYRARPATATVGQK 60
hFg Bβ chain     -------QGVNDNEEGFFSARGHRPLDKKREEAPSLRPAPPPISGGGYRARPAKAAATQK 53
                        :* :*. :  :.****** ***:********.**************.*:. **

bFg Bβ chain     KVERKPPDADGCLHADPDLGVLCPTGCKLQDTLVRQERPIRKSIEDLRNTVDSVSRTSSS 120
hFg Bβ chain     KVERKAPDAGGCLHADPDLGVLCPTGCQLQEALLQQERPIRNSVDELNNNVEAVSQTSSS 113
                 *****.***.*****************:**::*::******:*:::*.*.*::**:****

bFg Bβ chain     TFQYITLLKNMWKGRQNQVQDNENVVNEYSSHLEKHQLYIDETVKNNIPTKLRVLRSILE 180
hFg Bβ chain     SFQYMYLLKDLWQKRQKQVKDNENVVNEYSSELEKHQLYIDETVNSNIPTNLRVLRSILE 173
                 :***: ***::*: **:**:***********.************:.****:*********

bFg Bβ chain     NLRSKIQKLESDVSTQMEYCRTPCTVTCNIPVVSGKECEKIIRNEGETSEMYLIQPEDSS 240
hFg Bβ chain     NLRSKIQKLESDVSAQMEYCRTPCTVSCNIPVVSGKECEEIIRKGGETSEMYLIQPDSSV 233
                 **************:***********:************:***: ***********:.*

bFg Bβ chain     KPYRVYCDMKTEKGGWTVIQNRQDGSVDFGRKWDPYKQGFGNIATNAEGKKYCGVPGEYW 300
hFg Bβ chain     KPYRVYCDMNTENGGWTVIQNRQDGSVDFGRKWDPYKQGFGNVATNTDGKNYCGLPGEYW 293
                 *********:**:*****************************:***::**:***:*****

bFg Bβ chain     LGNDRISQLTNMGPTKLLIEMEDWKGDKVTALYEGFTVQNEANKYQLSVSKYKGTAGNAL 360
hFg Bβ chain     LGNDKISQLTRMGPTELLIEMEDWKGDKVKAHYGGFTVQNEANKYQISVNKYRGTAGNAL 353
                 ****:*****.****:*************.* * ************:**.**:*******

bFg Bβ chain     IEGASQLVGENRTMTIHNSMFFSTYDRDNDGWKTTDPRKQCSKEDGGGWWYNRCHAANPN 420
hFg Bβ chain     MDGASQLMGENRTMTIHNGMFFSTYDRDNDGWLTSDPRKQCSKEDGGGWWYNRCHAANPN 413
                 ::*****:**********.************* *:*************************

bFg Bβ chain     GRYYWGGAYTWDMAKHGTDDGVVWMNWQGSWYSMKKMSMKIRPYFPEQ 468
hFg Bβ chain     GRYYWGGQYTWDMAKHGTDDGVVWMNWKGSWYSMRKMSMKIRPFFPQQ 461
                 ******* *******************:******:********:**:*
```

C

```
bFg γ chain     YVATRDNCCILDERFGSYCPTTCGIADFLNNYQTSVDKDLRTLEGILYQVENKTSEAREL 60
hFg γ chain     YVATRDNCCILDERFGSYCPTTCGIADFLSTYQTKVDKDLQSLEDILHQVENKTSEVKQL 60
                *****************************..***.*****::**.**:********.::*

bFg γ chain     VKAIQISYNPDQPSKPNNIESATKNSKSMMEEIMKYETLISTHESTIRFLQEVYNSNSQK 120
hFg γ chain     IKAIQLTYNPDESSKPNMIDAATLKSRKMLEEIMKYEASILTHDSSIRYLQEIYNSNNQK 120
                :****::****:.**** *::** :*:.*:*******: * **:*:**:***:****.**

bFg γ chain     IVNLRDKVVQLEANCQEPCQDTVKIHDVTGRDCQDVANKGAKESGLYFIRPLKA-KQFLV 179
hFg γ chain     IVNLKEKVAQLEAQCQEPCKDTVQIHDITGKDCQDIANKGAKQSGLYFIKPLKANQQFLV 180
                ****::**.****:*****:***:***:**:****:******:******:**** :****

bFg γ chain     YCEIDGSGNGWTVFQKRLDGSLDFKKNWIQYKEGFGHLSPTGTGNTEFWLGNEKIHLIST 239
hFg γ chain     YCEIDGSGNGWTVFQKRLDGSVDFKKNWIQYKEGFGHLSPTGT--TEFWLGNEKIHLIST 238
                *********************:*********************  ***************

bFg γ chain     QSSIPYVLRIQLEDWNGRTSTADYASFKVTGENDKYRLTYAYFIGGDAGDAFDGYDFGDD 299
hFg γ chain     QSAIPYALRVELEDWNGRTSTADYAMFKVGPEADKYRLTYAYFAGGDAGDAFDGFDFGDD 298
                **:***.**::************** ***  * ********** **********:*****

bFg γ chain     SSDKFFTSHNGMQFSTWDSDNDKYDGNCAEQVGIGWWMNKCHAGHLNGVYYQGGTYSKTS 359
hFg γ chain     PSDKFFTSHNGMQFSTWDNDNDKFEGNCAEQDGSGWWMNKCHAGHLNGVYYQGGTYSKAS 358
                .*****************.****::****** * ************************:*

bFg γ chain     TPNGYDNGIIWATWKSRWYSMKKTTMKIIPLNRLAIGEGQQHQLGGAKQVGVEHHVEIEY 419
hFg γ chain     TPNGYDNGIIWATWKTRWYSMKKTTMKIIPFNRLTIGEGQQHHLGGAKQVRPEHPAETEY 418
                ***************:**************:***:*******:*******  ** .* **

bFg γ chain     D-------- 420
hFg γ chain     DSLYPEDDL 427
                *
```

Figure S1. Sequence alignments between bFg and hFg for Aα chain (A), Bβ chain (B), and γ chain (C), respectively. In each alignment, the first and second lines represent the sequences of bFg and hFg, respectively. The third line indicates results of the comparison. Asterisks indicate identical amino acids, colons indicate those possessing similar properties, and dots indicate those possessing moderately similar properties.

Figure S2

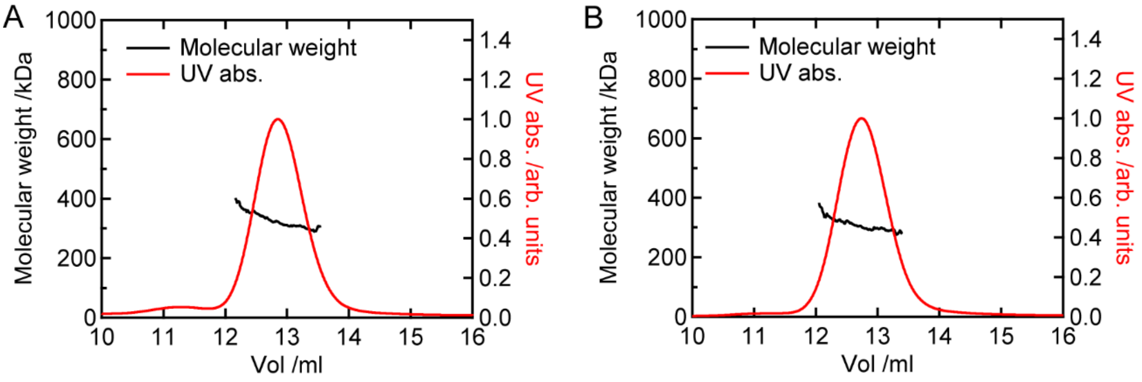

Figure S2. SEC-MALS results of bFg (A) and hFg (B), respectively. Both shows single peak possessing molecular weight close to their monomer (~340 kDa).

Figure S3

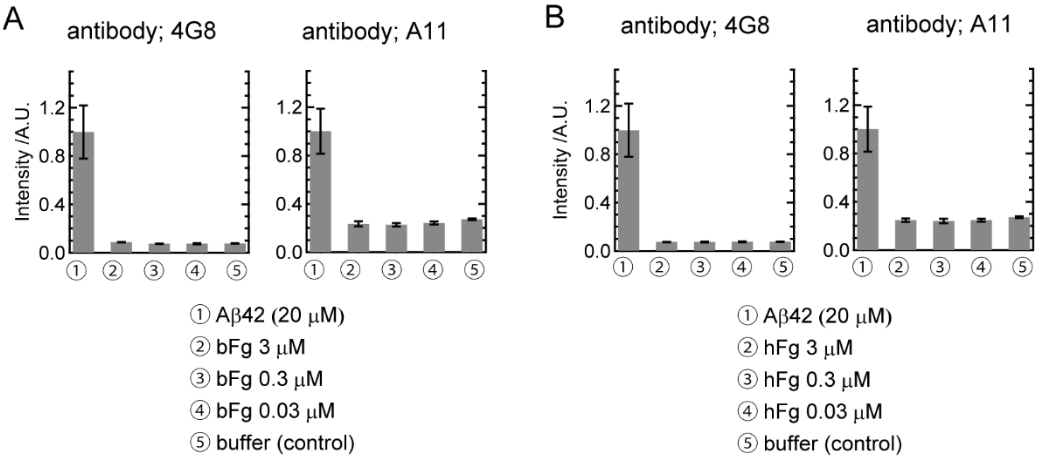

Figure S3. Dot blot results of bFg (A) and hFg (B). As controls, Aβ42 and buffer were also performed.

Figure S4

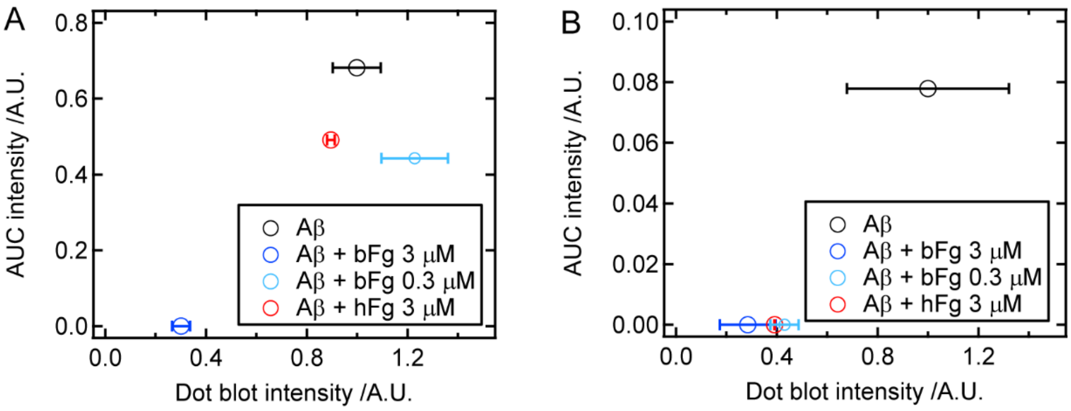

Figure S4. Correlations between dot blot and AUC intensities for Aβ monomers (A) and oligomers (B), respectively. Each sample is highlighted by a different color.

Figure S5

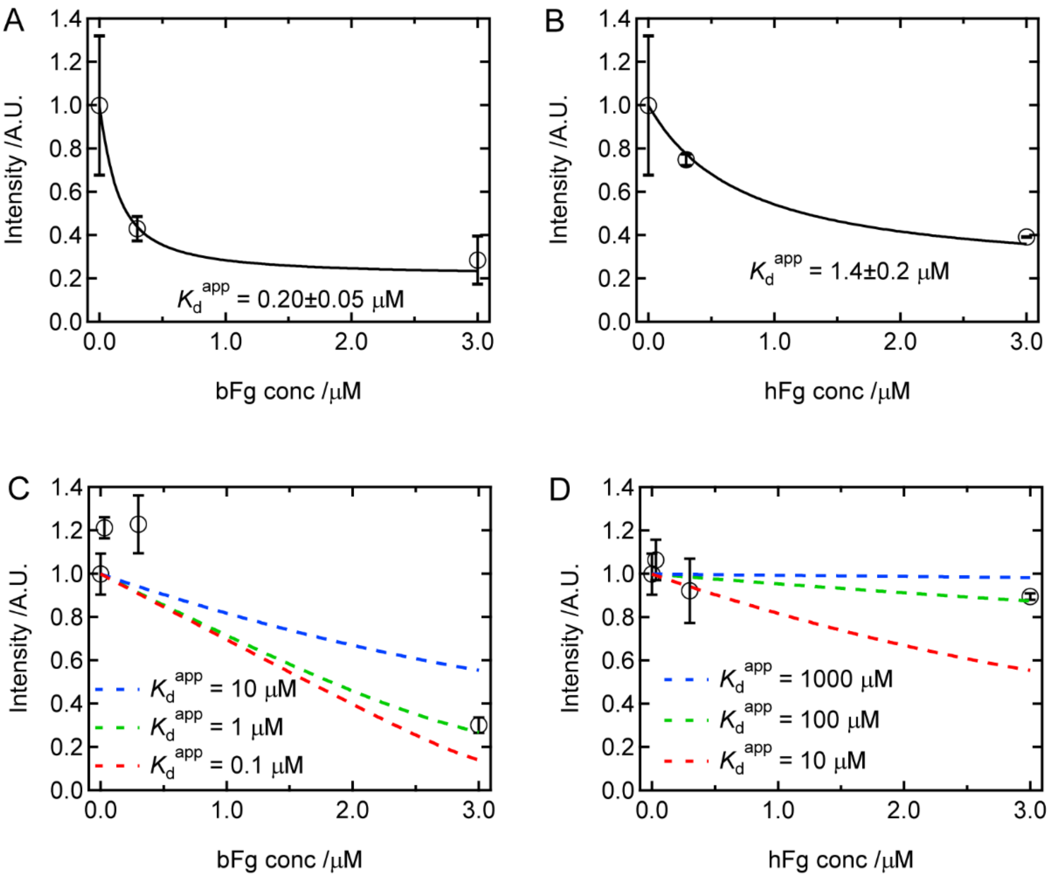

Figure S5. Analysis of dot blot results for A11 (A and B) and 4G8 (C and D), respectively. The spot intensity for A11 in the presence of bFg (A) or hFg (B) was fitted using the binding equation (eq. S6), obtaining the $K_{\mathrm{d}}^{\mathrm{app}}$ values. The spot intensity for 4G8 in the presence of bFg (C) or hFg (D) was simulated using the same binding equation to estimate the $K_{\mathrm{d}}^{\mathrm{app}}$ values.
